# The Pauper Sick in London

**Published:** 1898-09-10

**Authors:** 


### THE PAUPER SICK IN LONDON.

According to the English Poor Law, every pauper has a right to food and shelter and to proper medical attendance during illness, so, perhaps, it would be wrong to include the benefits offered to the sick poor by the Poor Law infirmaries among the charities of London. It is impossible, however, to gain an accurate conception of what is being done for the poor of the metropolis without a proper consideration of the large and rapidly-growing accommodation which is provided in these infirmaries, especially since, under modern conditions, they are in most cases completely separated from the workhouses, and, so far as their internal arrangements are concerned, can only be compared with general hospitals.

In all the workhouses of London, as in those of the provinces, there are large numbers of aged and infirm people, a considerable proportion of whom are really invalids. These, however, unless they are bed-ridden, are not included among the sick for whom alone the separate infirmaries are intended.

In considering the meaning of the following list, it must, then, be remembered that the accommodation there mentioned includes only what is intended for the "sick and bed-ridden."

The following is a list of the separate infirmaries under the Poor Law in London, with the number of beds provided by each:—

| | Number of beds. |
|---|---|
| Bethnal Green Infirmary ... ... ... | 650 |
| Camberwell Workhouse Infirmary ... ... | 451 |
| Central London Sick Asylum... ... ... | 264 |
| Chelsea Workhouse Infirmary ... ... | 360 |
| City of London Union Infirmary ... ... | 645 |
| Fulham Union Infirmary ... ... ... | 500 |
| Greenwich Union Infirmary ... ... ... | 400 |
| Hackney Union Infirmary ... ... ... | 600 |
| Hampstead Workhouse Sick Wards... ... | 184 |
| Holborn Union Infirmary ... ... ... | 617 |
| Lambeth Workhouse Infirmary ... ... | 541 |
| Lewisham Infirmary ... ... ... ... | 250 |
| Mile End Infirmary ... ... ... ... | 469 |
| Paddington Infirmary... ... ... ... | 284 |
| Poplar and Stepney Sick Asylum ... ... | 770 |
| St. George's Union Infirmary ... ... | 776 |
| St. George-in-the-East Parish Infirmary ... | 394 |
| St. Mary Abbott's (Kensington) Infirmary ... | 667 |
| St. Mary's (Islington) Infirmary ... ... | 680 |
| St. Marylebone Infirmary ... ... ... | 744 |
| St. Olave's Union Infirmary ... ... ... | 640 |
| St. Pancras Infirmary ... ... ... ... | 550 |
| St. Saviour's Union Infirmary ... ... | 786 |
| Shoreditch Infirmary ... ... ... ... | 472 |
| Wandsworth and Clapham Union Infirmary | 633 |
| Whitechapel Infirmary ... ... ... | 590 |
| Woolwich Union Infirmary ... ... ... | 288 |
| Total ... ... ... ... | 14,205 |

Thus we have in London a total of 27 large hospitals under the Poor Law containing in the aggregate no less than 14,205 beds for the sick, without including the hospitals of the Metropolitan Asylums Board, or the beds provided for the insane, the imbecile, the aged, or the merely infirm. Nor is this by any means a stationary figure. Before long it is certain that something will have to be done to improve the condition of the aged and infirm who are at present lodged in the workhouses; for while improved classification will, it is to be hoped, do something to make the conditions of life in the workhouse less attractive than they seem to be to the lazy able-bodied, it may be fairly expected, on the other hand, to do much to alleviate the lot of the respectable poor who, from mere age and misfortune have been driven to live out their days in the workhouse; so that in addition to the new infirmaries which are now arising in various quarters, we must not be surprised to find considerable extensions going on in the ordinary workhouse buildings. According to *London*, the following schemes for workhouse and infirmary extension are proposed or in progress:—

Hampstead, about £30,000 in workhouse extension.

Islington, £250,000 on a new infirmary.

St. Olave's, £164,000 on a new workhouse at Ladywell.

St. Saviour's, Southwark, is seeking a site on which to erect a new workhouse.

Bethnal Green, about £200,000 on a new infirmary, which will be opened before the end of the year; in addition to this, new casual wards are proposed, for which a site has been already secured.

Chelsea is about to build a new workhouse and to spend about £80,000 in extending the present building.

St. George's Union is also building extensions.

Marylebone has just opened extensions of its workhouse which have cost £50,000.

The Strand Union is adding to its accommodation in Drury Lane.

Wandsworth, besides expending £12,000 on a nurses' home, is contemplating erecting a new administrative block.

Camberwell Workhouse is being enlarged at an expense of £6,000.

Poplar Sick Asylum District is spending £10,000 on extension.

Whitechapel is adding another wing to its infirmary.

Paddington has purchased a site for a new workhouse; and

Mile End is expending £71,000 on cottage homes.

These figures seem very large, but a comparison of the number of beds in the Poor Law infirmaries with the mean number of indoor paupers (excluding vagrants and insane) in the metropolis shows how very considerable a proportion, very nearly a quarter, in fact, of all who receive indoor relief are really ill. Perhaps the most striking fact which is laid bare by a comparison of the returns of pauperism during the past thirty years is the very marked decrease, both in actual number and in ratio to population, of outdoor pauperism.

But along with this a steady increase is to be observed, in both numbers and ratio, of the indoor paupers. It seems probable, however, that this is to a considerable extent the result of the larger use of the Poor Law infirmaries by the sick poor. The overcrowding of the homes of the poor, the breaking up of family ties, due to increased facilities for locomotion, and the improved accommodation provided in the infirmaries, all tend to fill these buildings, not as they used to be filled with mere chronic ailments or modes of dying, but with acute diseases very much the same in kind as those which gain access to our general hospitals. So far as the increase of indoor pauperism is due to this cause, it is of course apparent only, and is not a matter for anxiety. The most alarming feature in the returns of indoor pauperism during recent years has been the increase in the number of able-bodied paupers who have accepted the workhouse rather than continue the effort to earn a living. The old and the feeble have done their work in life; they have indeed probably worked as well as any of us, and they deserve all that we can give them. As for the sick, who account for a good deal of the indoor pauperism, it is an economic advantage to the country that they should be restored to health as soon as possible. But with the able-bodied idle we have no patience. They are mere parasites, living on the good nature of the community, and should be eliminated as rapidly as possible.

